# Waist-Bands for Fat Men

**Published:** 1921-04-23

**Authors:** 


### Waist-Bands for Fat Men.

### A CASE FOR EXPERIMENT.

In the monthly publication which portrays the activities of the Industrial Welfare Society Dr. E. Halford Ross quotes from *The Times* what he describes as a very interesting and curious observation. A manual worker found that his energy failed gradually at his work until it was suddenly regained on joining the Army, during strenuous war life. On his return to his former civil employment he determined on and maintained hard exercise, with a view to retaining his war fitness. But, in spite of his exercise and determination, his energy failed him, and he sought the reason. Then someone told him that he was getting fat. So he decided to curb his fatness with a belt—a leather "Sam Browne" belt—drawn tight during working hours. The manual worker regained his fitness—which is the beginning and the end of the story.

But there is a moral, which is, according to the original teller of this story, that a belt on the belly forces breathing by the chest, which Nature meant us to breathe with, and that our abdominal muscles were never intended to perform the major part of our respiration, as we have allowed them to do. The theory of the "Sam Browne" belt fascinates, and at the same time, apparently, slightly puzzles Dr. Ross. In his first consideration of the matter he seems to support it, on the ground that human beings were originally intended to walk and work on "all fours," but that in the upright posture which we have chosen to adopt gravity brings into play the abdominal muscles, instead of the back and thorax, to drive the great chest pump. If, therefore, we curb the abdominal muscles with a narrow belt our chest muscles will tend to recover their aptitude, and, as in the case cited, we shall regain our fitness. There is an old Scotch riddle which asks why a fat man wears a plaid waistcoat, and the answer is that it puts a check on the stomach. But civilised women have worn corsets for generations, and it is doubtful whether they breathe better than men. Yet a corset can hardly be termed a belt, for it usually reaches above the lower ribs, thus impeding circulation. A woman athlete discards her corset for the championship, whereas a man tightens his belt, which, as Dr. Ross says, "knocks the bottom out of that argument." Again, the writer is informed that M. de Reske advises his pupils to wear a narrow belt and abdominal pad to improve voice production, which is dependent upon the bellows action of the chest. The conclusion is that the experience of the gentleman with the "Sam Browne" belt is interesting, but of itself proves nothing. There must be thousands of observations of the same kind to test the soundness of the theory, and there is no reason why ten thousand enthusiastic and strenuous manual workers with a slight tendency to embonpoint should not band themselves into a society to demonstrate the value of a tight girdle. "Leather straps are cheap," and harmless; and if they turn out to be the sovereign remedy for fatness the large-scale experiments will certainly have been worth while, in spite of the temporary injury done to the purveyors of quack medicines for reducing adipose tissue.

